# Establishing a sustainable quality management system in a public clinical laboratory: The case of Mbagathi Hospital Laboratory in Kenya

**DOI:** 10.4102/ajlm.v15i1.3032

**Published:** 2026-06-05

**Authors:** Jully A. Okonji, Martin K. Maitima, Peter K. Kariuki, Micheal Mugerwa, Romeo W. Kithuka, Maria Landron, Peter L. Nyanga, Bonventure Juma, Adolfo Lara, Rufus Nyaga, Elizabeth Hunsperger, Edwin Ochieng, Lucy Maryogo-Robinson, Naomi Lucchi

**Affiliations:** 1 Global Health Program, Association of Public Health Laboratories, Nairobi, Kenya; 2 Medical Laboratory, Nairobi County Laboratory, Nairobi, Kenya; 3 Medical Laboratory, Mbagathi Hospital Laboratory, Nairobi, Kenya; 4 Global Health Program, Association of Public Health Laboratories, Bethesda, Maryland, United States; 5 Ministry of Health, National Public Health Laboratories, Nairobi, Kenya; 6 Division of Global Health Protection, U.S. Centres for Disease Control and Prevention (CDC), Nairobi, Kenya; 7 Division of Global Health Protection, U.S. Centres for Disease Control and Prevention (CDC), Atlanta, Georgia, United States

**Keywords:** sustainability, resource limited settings, quality management, health facility, leadership commitment, facility focused mentorship

## Abstract

**Background:**

Between 2021 and 2023, the Association of Public Health Laboratories, in collaboration with the US Centers for Disease Control and Prevention in Kenya, initiated a laboratory improvement programme at the Mbagathi Hospital Laboratory by establishing a molecular assay to respond to the SAR-CoV-2 outbreak in the country and a quality management system (QMS) to improve the quality of laboratory services and processes.

**Intervention:**

Interventions were implemented through the procurement of molecular equipment, supplies, and staff capacity development through training and mentorship. Baseline, midterm, and end-term assessments were conducted, and data were collected using the World Health Organization Regional Office for Africa, Stepwise Laboratory Improvement Program Towards Accreditation tool, version 2015. Data were collected, analysed, and presented in graphs and tables.

**Lessons learnt:**

Transitioning to a self-sustaining model requires both financial autonomy and decentralised leadership. Empowering section heads, as quality leads, ensured operational continuity, even during high-pressure outbreaks. However, significant constraints included intermittent reagent stock-outs due to global supply chain disruptions and high personnel workload during peak pandemic waves. These were mitigated through standardised clinic-laboratory communication and prioritised local service contracts for equipment maintenance.

**Recommendations:**

To achieve resilient accreditation, public health facilities should move towards integrated financial models that leverage locally generated revenue. Institutionalising QMS within core hospital governance, rather than as a donor-dependent project, is essential for long-term sustainability in resource-constrained settings.

**What this study adds:**

While previous literature has established that QMS improves service delivery, our manuscript is unique in providing a documented roadmap for turning a diagnostic laboratory into a revenue-generating entity that ensures its own supply chain stability. Mbagathi Hospital now serves as a national benchmark, with other county hospitals seeking to emulate its model of efficient resource utilisation.

## Background

The need for a system-thinking approach when implementing laboratory-strengthening activities remains important, especially amid current global disease epidemiology, the ever-changing political landscapes, funding priorities, and the double burden of economic needs and infectious diseases, among others. Additionally, it has long been recognised that quality-assured functional laboratory services are needed for accurate diagnosis of infectious diseases like mpox (formerly monkeypox), severe acute respiratory syndrome coronavirus 2 (SARS-CoV-2), Ebola, and for current and emerging disease threats, including antimicrobial resistance, in malaria and *Mycobacterium tuberculosis*.^1,2,3^

In March of 2021, the Association of Public Health Laboratories (APHL), in collaboration with the U.S. Centers for Disease Control and Prevention (CDC)-Kenya, established a quality-assured molecular testing laboratory at the Mbagathi Hospital Laboratory (MHL) to respond to the demand for SARS-CoV-2 testing. As part of the laboratory system strengthening, a robust quality management system (QMS) was concurrently initiated to support International Standards Organization (ISO) 15189 accreditation. The MHL is a county referral hospital with clinical diagnostic services like bacteriology, molecular biology, serology, haematology, clinical chemistry, cytology, phlebotomy, and blood transfusion, and a general scope of 64 diagnostic tests. The hospital has a patient load of approximately 30 000 persons per month, with approximately 20 000 patients receiving services from the laboratory. The laboratory was originally enrolled in the Stepwise Laboratory Improvement Program Towards Accreditation (SLIPTA)^4^ in 2011–2013 and had attained a maximum of STAR 2, symbolising stepwise improvement towards achieving accreditation. However, the laboratory QMS had stagnated, partly due to a suboptimal commitment from both laboratory and hospital leadership, and the laboratory was dropped by the supporting partner from the list of laboratories under SLIPTA mentorship. Therefore, there was a need to establish a strong, sustainable QMS in the MHL to support the laboratory activities and services. The implemented QMS was envisioned to operate using the available and existing laboratory human resources and infrastructure at MHL.

We report on the results and benefits following the establishment of a quality-assured molecular laboratory as well as how QMS implementation led to streamlining the rest of the laboratory processes.

## Description of the intervention

### Ethical considerations

An application for ethical approval was made to the Mbagathi Hospital, Health, Wellness and Nutrition Services, Nairobi City County on 15 October 2025. The Mbagathi County Referral Hopsital issued an ethics waiver for the study because the research was undertaken within the framework of the hospital’s accredited laboratory.

Laboratory programmatic data from the SLIPTA tool was used, and no human subject information was collected. Laboratory QMS data were stored and handled with restricted access except for the respective laboratory staff, the hospital superintendent, APHL mentors, and CDC technical advisors.

### Establishment of a molecular laboratory

Between March 2021 and September 2022, the MHL was reorganised to accommodate a molecular section for SARS-CoV-2 testing.The novel molecular section was refurbished to enable a unidirectional workflow and installation of molecular equipment, including a real-time polymerase chain reaction analyser, refrigerated centrifuge, heating block, polymerase chain reaction workstation, freezer, vortex mixer, and autoclaves. Molecular reagents and commodities were purchased and delivered. The staff were trained on using the new test workflows, equipment verification, and QMS consistent with the ISO 15189 requirements.^5^

### Laboratory quality management system implementation using facility-focused mentorship

The laboratory staff were trained by APHL mentors using facility-focused mentorship on laboratory work processes. Staff competency was determined before and after training. A meeting was held for the hospital management team to better understand the need for QMS sensitisation and the hospital management’s commitment. A mentorship schedule was developed that accommodated all 12 quality essential areas for the laboratory to attain ISO accreditation status. The mentorship visits included onsite training on ISO 15189 implementation, risk assessment and risk management, internal audits, method validation and verifications, and customer care services. The mentorship targeted hospital management, laboratory leadership, and laboratory staff. The APHL mentors spent 1–2 weeks every 2 months during the intervention period and worked closely with the quality officer to address the gaps identified during this process. Additionally, the mentors worked closely with the hospital leadership to provide additional resources and for the laboratory staff to develop work schedules that had well-defined, measurable, achievable, and time-bound goals derived from the 12 Quality System Essentials (QSEs) in the World Health Organization Regional Office for Africa SLIPTA checklist.

### Laboratory quality management system implementation sustainability approach

The APHL adopted QMS and laboratory services sustainability approaches by addressing four key pillars: (1) human resources, (2) financial resources, (3) infrastructure (laboratory workspaces, storage, electricity supply and water), and (4) laboratory information management system. In addition, the interaction between these approaches was assessed and addressed during the mentorship to ensure efficiency and effectiveness. A laboratory Strengths, Weakness, Opportunities, and Threats assessment was conducted by the mentors and outcomes shared with both the laboratory team and the hospital management. Priority areas that required action and commitment from both parties were discussed during feedback sessions. Clear objectives were discussed and included establishing a quality-assured molecular laboratory run by the laboratory staff, improving laboratory quality services beyond the molecular work, and identifying opportunities for laboratory improvement across technical and management-related processes, including the development of lab standard operating procedures.

### Human resource capacity

Various factors were considered to understand the human resource capacity. This included the number of laboratory staff, their skills and specialties (whether utilised or not), the ability of the laboratory staff to manage their time across the various departments, the laboratory leadership structure, and governance capacity to support sustainability.

The laboratory management underwent strategic restructuring to ensure operational continuity and QMS sustainability. This included a multi-tiered leadership hierarchy comprising a laboratory manager, quality officer, and safety officer, each supported by a designated deputy. Significantly, section heads were formally empowered with management and quality oversight responsibilities. This intentional design served as a robust succession plan: By training section heads to act as functional quality and laboratory managers within their respective units, the laboratory eliminated leadership gaps and ensured that technical and quality activities remained uninterrupted, even in the absence of senior management.

The laboratory organogram was communicated to staff, and each staff member’s job description was clear, outlining responsibilities, delegated roles, and reporting lines, bringing clarity in personnel management. The designee for any leadership position was expected to perform at the same level as the primary position holder in terms of service delivery. Occasionally, the laboratory leadership appointed lower-level staff to managerial roles with guidance from the laboratory manager as part of staff capacity leadership mentorship.

### Financial resource capacity

Laboratory resource capacity was defined as current, potential, and future capacities and opportunities. Monetary resources included hospital-allocated funding for laboratory operations, alternative financial resources like loans, laboratory revenue collection, and, where feasible, occasional donations from the central government or implementing partners, resources, and a potential resource partner.

#### Infrastructure

Infrastructure was defined as the laboratory environment where activities are performed, the status of the equipment used in laboratory processes, the laboratory infrastructural organisation, and its influences on laboratory processes. This also included electricity needs, water, lighting, and safety for both patients and staff and other laboratory users.

#### Information management

Information management was defined as how the laboratory collects, sends, receives, and communicates information. In addition, this included the use of information to guide projection decisions, and use of such information to improve laboratory services.

### Laboratory assessments

Baseline, mid-term, and end-term assessments were performed before, during, and after APHL/CDC’s engagement with MHL. Assessment data were collected using the WHO AFRO SLIPTA checklist version 2.0 (2015) and stored in an Excel sheet 2010 version (Microsoft Corporation, Redmond, Washington, United States), and data on laboratory performance were collected on workload, laboratory revenue, frequency of joint clinical laboratory interface meetings during ward rounds or continuous medical education series, and other performance quality indicators. These data were recorded on QMS tools as part of a continuous laboratory improvement programme, and a summary was captured in an Excel worksheet. The baseline assessment was conducted in March 2021, while the midterm and end-term assessments were conducted in October 2021 and December 2023.

## Data analysis

The primary analysis focused on laboratory improvement process trends from baseline to end-term based on the 12 QSEs described in the SLIPTA^5^ model using data collected between January 2021 and December 2023. Quality System Essentials performance was presented graphically and in tables. An average performance of the individual QSE was obtained. Data were analysed using Excel and SPSS version 20.0 (IBM Corporation, Armonk, New York, United States). A paired *t*-test was used to determine the mean statistical difference between baseline and end-term. Improvements in SLIPTA standard deviation difference of > 25% were considered significant (*p* < 0.0001). As per the SLIPTA guidance, star ratings were awarded according to overall SLIPTA score: < 55% = 0 Star, 55% – 64% = 1 Star, 65% – 74% = 2 Stars, 75% – 84% = 3 Stars, 85% – 94% = 4 Stars and > 95% = 5 Stars.

### Observed characteristics of Mbagathi Hospital Laboratory before and after the mentorship

Before mentorship, there was no evidence of QMS implementation. After the mentorship, MHL and the mentor developed and implemented guidelines for technical and management procedures and tools for collecting records, as described in Table 1. Of the total 63 tests performed in the laboratory, 45 (70%) were enrolled in a proficiency testing panel with a consistent performance of > 80% score and were included in the scope of accreditation.

**TABLE 1:** Characteristics of Mbagathi Hospital Laboratory Quality Management System implementation by December 2022, Kenya.

Characteristics	Proportions before the APHL/CDC programme mentorship			Proportions after APHL/CDC programme mentorship		
	*n*	*N*	%	*n*	*N*	%
**Documents (tools developed and implemented)**						
Policy guidance	1	3	33	3	3	100
Technical procedures	10	65	15	63	65	96
Management procedures	0	25	0	25	25	100
Forms for capturing records	20	102	20	102	102	100
**Laboratory discipline (no. of tests on the scope of accreditation) (*N* = 63)**						
Haematology (FBC) (16 test parameters)	0	16	0	16	16	100
Molecular biology	0	2	0	2	2	100
Clinical chemistry (19 test parameters)	0	19	0	19	19	100
Bacteriology (blood, stool, pus, urine, cerebral spinal fluid, and swab)	0	6	0	6	6	100
Parasitology (malaria microscopy)	0	3	0	1	3	33
Blood transfusion	4	4	100	4	4	100
HIV comprehensive care and care laboratory (TB and HIV)	0	2	0	2	2	100
Cytology	0	0	0	5	5	100
Immuno-assays (markers)	0	0	0	8	8	100
**External quality assessment (EQA) Enrolled (*n* = 45/65)**						
One-world accuracy	N/A	N/A	N/A	SARS-CoV-2		
World Health Organization	N/A	N/A	N/A	SARS-CoV-2		
KNEQAS-EQA provider – Government	N/A	N/A	N/A	TB, malaria, HIV serology, bacterial culture (swab, urine, stool, blood, pus, and CSF)		
KETON- EQA provider – Privately sourced	N/A	N/A	N/A	Full blood count (*n* = 16) and clinical chemistry (*n* = 19)		
**Laboratory legal subscription (actively paid annually)**						
Accreditation body subscriptions (twice annually) 2022/2023	N/A	N/A	N/A	2	-	100
Professional body (once annually) 2022/2023	N/A	N/A	N/A	2	-	100
EQA provider – KETON (thrice annually) 2022/2023	N/A	N/A	N/A	3	-	100
Sources of funding for laboratory service	Overall	-	-	-	-	-
Devolved government through its own revenue generated from the hospital through Facility Improvement Fund (FIF)	-	-	40	90% (hospital and county)		
Implementing partners (separate capital equipment investment)	-	-	59	HIV, TB, malaria programmes		
National government	-	-	1	Occasionally based on donations.		

%, Percentage; *n*, Number of observations; EQA, External Quality Assessment; KNEQAS, Kenya National Quality Assurance Service; MLS, Medical laboratory scientist; CSF, Cerebral spinal fluid; TB, *Tubercule bacilli*; FBC, Full blood count; APHL/CDC, Association of Public Health Laboratories/Centers for Disease Control; SARS-CoV-2, severe acute respiratory syndrome coronavirus 2; N/A, not applicable.

#### Financial resource

The laboratory receives its budget allocation from the devolved county government hospital’s quarterly budget programme generated through revenue collected for services offered. However, laboratory services are offered free to children under five, prisoners, and those who cannot afford payment, in accordance with the hospital policy. The MHL did not receive any substantive budget allocation before 2021. The laboratory lacked a structured method for collecting funds, resulting in a gap in its revenue-tracking system. Additionally, many processes needed to be improved to enhance efficiency. Before the mentorship, the laboratory collected a revenue of approximately 0.5 million Kenya shillings in a quarter, which was not sufficient to fund laboratory activities such as procurement of reagents, proficiency testing panel, or equipment preventive and corrective services. No budget was allocated to the laboratory from the hospital, and the procurement of laboratory supplies was greatly affected. On some occasions, the laboratory operated on loaned reagents from the supplier, but when this was not possible, it led to long reagent stock-outs and extended equipment downtime, affecting the service provision. There were insufficient resources to enrol laboratory tests on proficiency testing panels, except for those provided free through the National Public Health Laboratory, such as proficiency testing panels for tuberculosis GeneXpert, and HIV serology.

**FIGURE 1:**
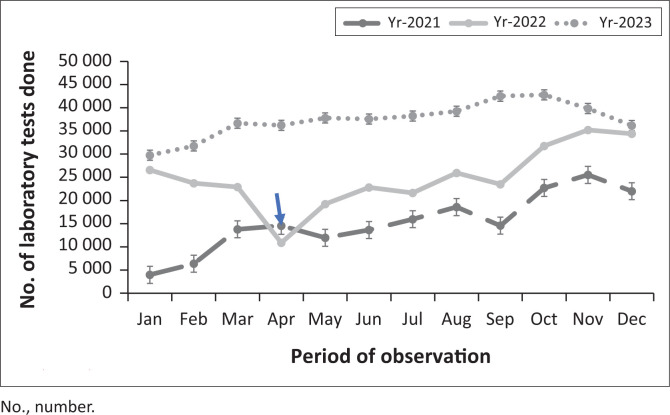
Number of laboratory tests conducted at the Mbagathi Hospital Laboratory in 2021, 2022, and 2023.

**FIGURE 2:**
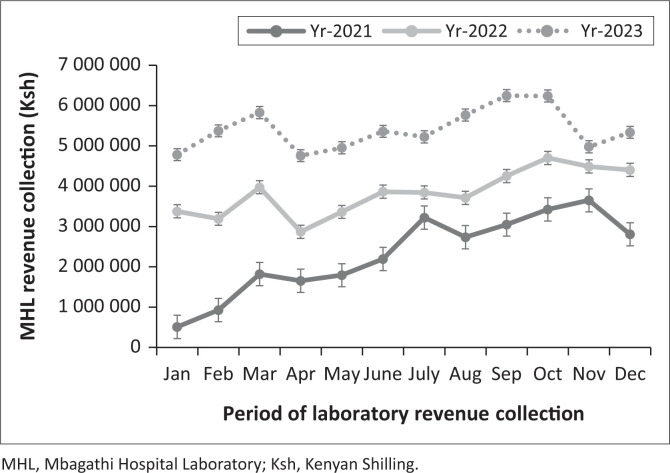
A line graph indicating the laboratory revenue collection over time for the period January–December 2021, 2022, and 2023 for Mbagathi Hospital Laboratory, Kenya.

**FIGURE 3:**
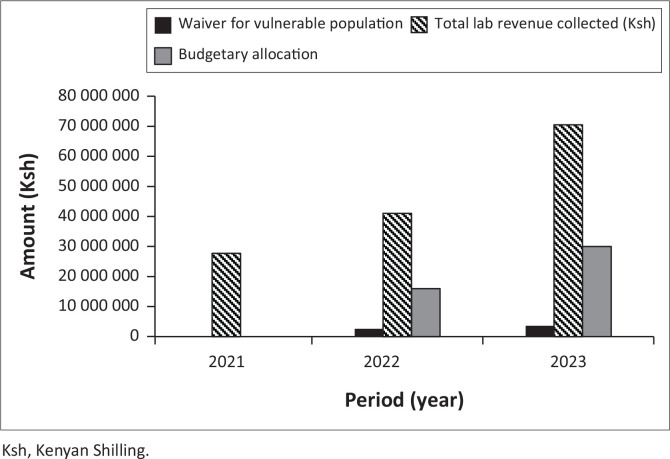
Budgetary allocation for the Mbagathi Hospital laboratory to run all the laboratory functions in 2021, 2022, and 2023.

There was an increase in the number of tests performed by MHL over time (Figure 1). The total number of laboratory tests performed in 2021 was 113 400 compared to 231 081 in 2022 and 446 428 in 2023. A drop in tests performed was observed in April, September, and December of 2021 and 2022, and was associated with some reagent stock-outs due to delays in payment of supplies. However, overall, there was an increase in the laboratory revenue collection over the same period (Figure 2). During the mentorship process, the laboratory and hospital leadership entered into an informal agreement stating that if improvements in laboratory service delivery and revenue collection are observed, the hospital would, in turn, reinvest the revenue collected for laboratory services to support the laboratory operations, including reagent and proficiency testing panels procurements. Strategies to increase laboratory revenue included, but were not limited to, introducing new test menus, such as cytology and microbiology, increasing staff training on QMS, and improving customer care. In 2 months, the laboratory doubled its revenue. We observed an increased engagement with hospital leadership in terms of support for equipment service contracts, procurements of essential laboratory reagents, and partly paying for accrued debt. The MHL did not receive any substantive budget allocation before 2021; however, the laboratory recorded an increase in its budget allocation to 16 million Kenya shillings (125 000 USD) in 2022 and then to 30 million Kenya shillings (234 375 USD) in 2023 (Figure 3).

#### Human resource

The laboratory had 23 technical staff in 2021, including two postgraduates, three graduates, and 18 undergraduates and diploma holders. During the mentorship, the hospital provided funds to hire additional staff on a short-term basis due to the workload. During this period, there was no staff attrition other than retirements, and an improvement in staff attitude towards QMS implementation and daily laboratory service delivery was observed. All staff attended laboratory meetings and participated in the training conducted onsite every month. Quality management system meetings were organised based on staff work schedules, and other forms of communication, such as WhatsApp groups, were utilised to pass information. Laboratory staff training included ISO 15189:2012 implementation, method validation and verification, risk assessment and risk management, and customer care. The hospital superintendent attended most of the laboratory training and made follow-up action plans, including providing resources when needed.

### Quality management system implementation performance at the Mbagathi hospital laboratory

**FIGURE 4:**
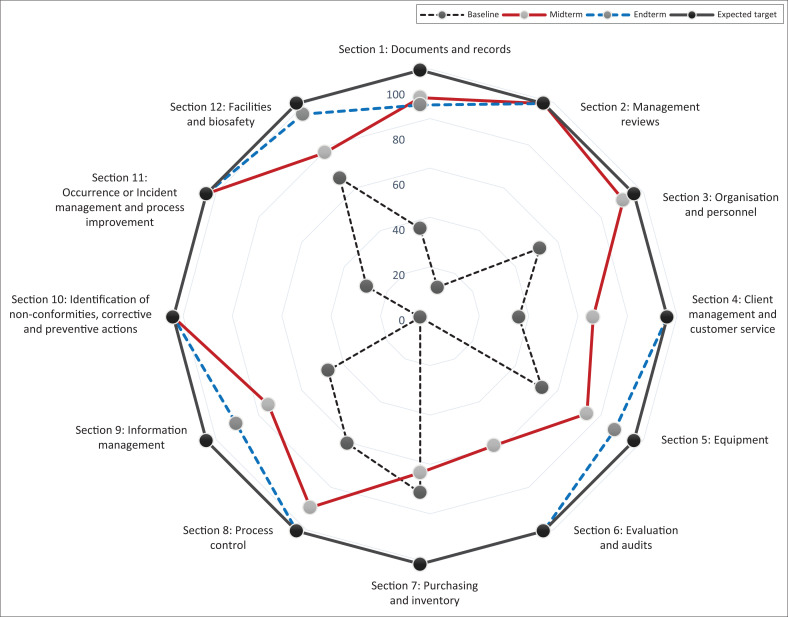
The Mbagathi Hospital Laboratory Quality Management Systems implementation performance at baseline (127/275; 46%, 0 Star, 2022), midterm (224/275; 81%, 3 Star, 2022), and end-term (260/275; 94%, 4 Star, 2022) between the observation period, January 2021 – December 2022, Kenya.

An average score of 46% (*n* = 127 of *N* = 275) was recorded during the baseline assessment, which improved to 95% during both midterm and end-term assessments (see Figure 4). Improvements were recorded in QSEs sections: Management review, evaluation, identification of non-conformities, and corrective and preventive actions when comparing baseline and end-term time points (see Table 2).

**TABLE 2:** Stepwise Laboratory Improvement Program Towards Accreditation baseline results versus end-term (post-intervention) outcome.

QMS implemented areas	Baseline outcome			Midterm outcome			End-term outcome			Improved percentage absolute difference from baseline to end-term
	*n*	*N*	%	*n*	*N*	%	*n*	*N*	%	%
**Section 1:** Documents and records	10	28	36	25	28	89	24	28	86	50
**Section 2:** Management review	2	14	14	14	14	100	14	14	100	86
**Section 3:** Organisation and personnel	12	22	55	21	22	95	22	22	100	45
**Section 4:** Client management and customer service	4	10	40	7	10	70	10	10	100	60
**Section 5:** Equipment	20	35	57	25	35	71	29	35	83	26
**Section 6:** Evaluation and audits	0	15	0	9	15	60	15	15	100	100
**Section 7:** Purchase and inventory	17	24	71	15	24	63	24	24	100	29
**Section 8:** Process control	19	32	59	29	32	91	32	32	100	41
**Section 9:** Information management	9	21	43	15	21	71	18	21	86	43
**Section 10:** Identification of non-conformities, corrective and preventive actions	0	19	0	19	19	100	19	19	100	100
**Section 11:** Occurrence/Incident management and process improvement	3	12	25	12	12	100	12	12	100	75
**Section 12:** Facilities and biosafety	28	43	65	33	43	77	41	43	95	30
Total score	127	275	46	224	275	81	260	275	95	49
**WHO AFRO SLIPTA Star score**	**0 Star**			**3 Star**			**4 Star**			**62**

WHO AFRO SLIPTA, World Health Organization Regional Office for Africa Stepwise Laboratory Improvement Program Towards Accreditation; QMS, quality management system.

Overall, baseline performance based on the WHO AFRO SLIPTA scores on QSEs analysed at three time points is presented in Figure 4. At baseline, the laboratory had a score of 127/275 (46%), equivalent to a 0 Star rating; at midterm, 224/275 (81%) points, equivalent to a 3 Star rating, and at the end of term, 260/275 (94%) points, equivalent to a 4 Star rating. Quality management system scores obtained at baseline, compared to midterm and end-term, demonstrated improvement. The MHL achieved ISO accreditation in December 2022; 15 months from initiation of the mentorship process to accreditation is considered a short time compared to other previously accredited laboratories.^6^ However, four areas of the QSE registered low progress in implementation: Information management, equipment management, evaluation and audits, and facility and safety. Laboratory mentorship was then focused on these areas to help the laboratory improve, including strengthening laboratory information systems and equipment interfacing. Overall, there was a statistically significant improvement from baseline to end-term outcomes across the QMS sections (paired *t*-test, *t*(11) = 7.47, *p* < 0.0001).

### Infrastructure

This capacity was essential for the smooth workflow in the laboratory as it influenced facility safety, accommodation, electricity needs, water, lighting, and safety for both patients and staff. During the intervention period, leaking sinks and broken windows were repaired. Surveillance cameras were installed for safety, and fire extinguishers were purchased and installed in the laboratory premises to ensure fire safety and risk management. The laboratory management ensured that the equipment was functional and serviceable. This led to continuous service provision to patients and other laboratory users through a constant supply of reagents and consumables, ensuring services were always available.

## Discussion

Overall, performance improved following 2 years of mentorship and evaluation. Through facility-focused mentorship, the Mbagathi laboratory received its ISO 15189 accreditation within 15 months compared to 24–37 months typically required to achieve accreditation by other public laboratories in Kenya.^7^ This accreditation status led to an increase in the uptake of laboratory services from within the hospital and from other external services. This observation is not unusual as QMS, or accreditation, has previously been shown to lead to an increase in laboratory services due to improvement in service delivery; however, for this facility, the increase in workload also led to an increase in revenue collection, as demonstrated in both Figure 1 and Figure 2.^3,7,8,9^ The establishment of the molecular laboratory elevated the laboratory status from Class D laboratory to Class F laboratory as per the Kenya Medical Laboratory Technologist and Technician Board rating signifying a change from a routine diagnostic laboratory to a referral laboratory.

The baseline assessment was utilised for corrective and preventive action. This led to streamlining laboratory services for pre-examination, examination, and post-examination processes leading to overall improvement in the quality of service provided. Additionally, improvement in the laboratory revenue collection led to the laboratory being allocated more funding every quarter. Initially, in 2021, the laboratory budget allocation was not clear as procurement of the services or commodities was done on a need-by-need basis, and many times, a push system was used in procuring laboratory reagents and commodities, leading either to less supply or oversupply of certain commodities thus expiry if not consumed in time. However, from September 2021 through 2023, the laboratory was involved in the hospital’s budget allocation committee and was able to defend or justify its budget allocation using data. With the increased laboratory budget allocation, there were very few instances of service halts and no reported reagent stock-outs. Additionally, this seamless budget allocation enabled the section heads, the quality officer, and the laboratory manager to work on the test projections and consumption rates for the various tests for evidence-based procurement planning.

Although the laboratory demonstrated exceptional performance in QMS implementation, some areas, such as information management, did not achieve the target outcome. The laboratory continued to use logbooks to manage its data, which was not desirable. The observed reduction in the number of laboratory services in April 2022 was associated with changes in the hospital leadership, which resulted in a delay in the disbursement of some resources during the transition period. Even though there was a reduction in some laboratory services, this was mostly associated with a delay in the disbursement of money owed by some vendors. However, this is not an abnormal occurrence in a transition period in any high leadership role. These occurrences and their effects were discussed in the management review meeting and identified as areas of improvement. This signifies the role of leadership and governance in the stability and sustainability of laboratory services.

The MHL has served as an example in Kenya, where other county hospital laboratories visit to learn how to establish a successful public clinical laboratory. The MHL laboratory has demonstrated that it is possible to sustain laboratory services through efficient resource utilisation and strong laboratory and hospital leadership and governance.

## Lessons learnt

The successful transition from donor dependency to a self-sustaining model was driven by laboratory leadership’s commitment and financial autonomy. By negotiating a strategic revenue-retention agreement with hospital management, the laboratory reinvested increased service income directly into QMS infrastructure and reagent procurement. This internal budgetary growth directly addressed the ‘funding cliff’ often associated with the conclusion of donor-funded projects. Furthermore, a decentralised leadership model empowered section heads, such as quality leads, ensuring operational continuity and strong staff buy-in. This structure maintained QMS integrity even during the high-pressure environment of the SARS-CoV-2 outbreak. Finally, establishing a formal clinic-laboratory interface working group ensured that laboratory data were effectively integrated into clinical decision-making, significantly reducing pre-analytical non-conformities through standardised sample-collection manuals and laboratory-led clinical rounds.

## Recommendations

To achieve sustainable accreditation in resource-constrained settings, laboratories must transition towards integrated financial models that leverage locally generated revenue. It is essential to institutionalise QMS within the hospital’s core governance framework rather than managing it as a peripheral, donor-dependent project. We recommend that public health facilities adopt multi-tiered leadership structures and formal revenue-sharing agreements to ensure that quality improvements remain resilient to fluctuating external funding climates. Additionally, the implementation of structured clinic-laboratory communication loops is recommended to harmonise clinical requirements with laboratory operations, ensuring that the QMS provides measurable value to patient care.
